# *Lactobacillus rhamnosus PL1* and *Lactobacillus plantarum PM1* versus placebo as a prophylaxis for recurrence urinary tract infections in children: a study protocol for a randomised controlled trial

**DOI:** 10.1186/s12894-020-00723-1

**Published:** 2020-10-23

**Authors:** Maria Daniel, Hanna Szymanik-Grzelak, Agnieszka Turczyn, Małgorzata Pańczyk-Tomaszewska

**Affiliations:** Department of Paediatric Nephrology, Medical University of Warsaw, Żwirki i Wigury 63A, 02-091 Warsaw, Poland

**Keywords:** Recurrent UTI, Children, Probiotics, Prophylaxis, RCT, *Lactobacillus rhamnosus PL1*, *Lactobacillus plantarum PM1*

## Abstract

**Background:**

Urinary tract infections (UTIs) are one of the most common bacterial infections in children. In children < 7 years of age, the prevalence of one episode of symptomatic UTI has been estimated at 3–7% in girls and 1–2% in boys, whereas 8–30% of them will have one or more episodes of UTI. The use of some probiotics appears to reduce the risk of recurrence of UTIs. Since the effects of probiotics are strain-specific, the efficacy and safety of each strain has to be assessed. The main aim of this study is to determine whether probiotics (containing *Lactobacillus rhamnosus PL1* and *Lactobacillus plantarum PM1)* therapy are effective in preventing UTI in children compared to placebo.

**Method:**

A superiority, double-blind, randomised, controlled trial is being conducted. One hundred and six patients aged 3 to 18 years with recurrent UTIs in last year (defined as: ≥ 2 episodes of UTI with acute pyelonephritis/upper UTI; or 1 episode of UTI with acute pyelonephritis and ≥ 1 episodes of UTI with cystitis/lower UTI; or ≥ 3 episodes of UTI with cystitis/lower UTI) or children with ≥ 1 infection in the upper urinary tract and ≥ 1 of recurrent UTIs risk factors (congenital anomalies of the kidney and urinary tract, constipation, bladder dysfunction, myelomeningocele, sexual activity in girls) will be randomly assigned to receive a 90-day prophylaxis arm (probiotic containing *L. rhamnosus PL1* and *L. plantarum PM1*) or a 90-day placebo arm. The primary outcome measure will be the frequency of recurrence of UTI during the intervention and in the period 9 months after the intervention.

**Discussion:**

The findings of this randomised controlled trial (RCT), whether positive or negative, will contribute to the formulation of further recommendations on prevention of recurrent UTIs in children.

***Trial registration number*:**

NCT03462160, date of trial registration 12th March 2018.

## Backround

Urinary tract infections (UTIs) are one of the most common bacterial infections in children. In children < 7 years of age, the prevalence of one episode of symptomatic UTI has been estimated at 3–7% in girls and 1–2% in boys, with 8–30% of them will have one or more recurrences of a UTI [1–3].

In previously published European and global guidelines, there have been no clear recommendation for prohylaxis of UTIs. According to the current recommendations, including the Polish Society of Paediatric Nephrology, antibacterial prophylaxis should be considered in children with congenital anomalies of kidney and urinary tract (CAKUT) with a history of UTIs [1, 2, 4–7].

Some randomised studies found no beneficial effect of antibiotic prophylaxis in decreasing the frequency of UTIs or preventing renal scarring. Furthermore, the antibiotic prevention strategies was associated with bacterial resistance [3, 8].

In recent years, we have observed an increasing interest in alternative methods of UTIs prevention, such as immunotherapy and probiotic therapy [9–12]. Use of some probiotics appears to reduce the risk of UTIs. Lee and et al. conducted randomised trial in children with persistent primary vescioureteral reflux (VUR) and with history of recurrent UTIs [13] and in children with VUR < 1 year old having experienced pyelonephritis [14], comparing the effect of *L. acidophilus* to a low-dose trimethoprim and sulfamethoxazole therapy (TMP/SMX). The effect on recurrent UTIs of probiotic and antibiotic therapy did not significantly differ [13, 14]. There are also some published trials with probiotics and their influence on decreasing UTIs in adult women [15–17]. In the up-to-date research, the methodology used varied considerably [18, 19]. Since the effect of probiotics are strain specific, the efficacy and safety of each strain has to be assessed.

This study products contains a specific combination of two bacterial strains *L. rhamnosus PL1* and *L. plantarum PM1*.

It is suggested that lactobacilli bacteria had a natural ability to move along gastrointenstinal tract to the rectum and anus and from where they migrate to the urethra and vagina [20, 21]. Lactic acid-producing bacteria may positively affect the urogenital microflora due to the strong adhesion to the epithelial urogenitaly tracts and displacing uropathogenic microorganism [22, 23]. Moreover, probiotics may inhibit the growth of pathogenic microorganism by producing substances, such as lactic and acetic acid, and bacteriocins [24, 25]. They may also prevent infections by immunomodulation [23, 26]. Some intervention studies have been reported if the administration of specific *Lactobacillus* strains can prevent UTIs [27, 28].

## Methods/design

### Trial objectives and hypothesis

The investigators aim to assess the effect and safety of administration of probiotic containing: *L. rhamnosus* PL1 with *L. plantarum* PM1 in prevention of recurrent UTIs in children. We hypothesise that study product is more effective than placebo in prophylaxis of UTIs in children.

The trial is registered at ClinicalTrials.gov (NCT03462160) and any significant changes will be included there.

### Trial design

This study is designed as a randomised, placebo-controlled, double-blind, superiority trial.

### Settings and participants

The study will be performed in paediatric units of the paediatric hospital and the nephrology outpatient clinic of the Medical University of Warsaw. The recruitment started in July 2018 and should be completed within the following 3 years.

### Eligibility criteria

Participants must fulfil all inclusion criteria to be recruited for the trial:

aged from 3 to 18 years; diagnosis of recurrent UTIs in the last year, defined as:

-  ≥ 2 episodes of UTI with acute pyelonephritis/upper UTI
- 1 episode of UTI with acute pyelonephritis and ≥ 1 episodes of UTI with cystitis/lower UTI
-  ≥ 3 episodes of UTI with cystitis/lower UTI [29].

or 1 infection in the upper urinary tract and ≥ 1 of UTIs risk factors: CAKUT, constipation, bladder dysfunction, myelomeningocele, sexual activity in girls; ≥ 1 episode of urinary tract infection in the last 6 months; signed informed consent.

### Exclusion criteria

Will include the following: intake of probiotic preparations for ≥ 1 month in the last 3 months; antibiotic use within last month due to any reason, known allergy to the study products, immunosuppression therapy, disease with immune deficiency, central catheter and children with other coexisting infections, e.g. meningitis, sepsis, pneumonia, otitis.

### Interventions

All participants will be provided with probiotics containing *L. rhamnosus PL1* with *L. plantarum PM1* or placebo. The placebo powder consists of a mixture of potato maltodextrin, glucose, arabic gum, pectin and silicon dioxide. The formulation is identical with the active products but without *L. rhamnosus PL1* and *L. plantarum PM1*. The appearance of the placebo will be comparable to the powder containing probiotics. Placebo as the gold standard for assessing the effectiveness of a new treatment was chosen as the comparator in our trial.

The study products (probiotic and placebo) will be manufactured and supplied by Miralex (Pila, Poland) free of charge. The manufacturer will not take part in conception and protocol preparation, design and conduct study, or in the process of analysing and interpretation of the data.

### Study procedure

Oral and written information will be given to parents of each participants and children > 16 years old. Participants will be randomised during hospitalisation or visit at outpatient clinic. Eligible patients will receive *L. rhamnosus PL1* with *L. plantarum PM1* at a dose of 10^9^ CFU (2 g) each or placebo, orally, once daily, in the evening during a meal, after dissolving the powder in lukewarm water. The probiotics or placebo will be administered for 90 days. Throughout the study period caregivers will record the UTI. Caregivers will have the right to withdraw a participating child from the study at any time and they will not be obliged to give reasons for this decision and this will not affect subsequent medical care.

In the event of UTI, the proper treatment will be implemented [6].

### Follow-up

All study participants will be followed up directly after intervention and 3, 6 and 9 months after the intervention.

### Compliance

Face-to-face interview with patients and/or caregiver and through daily patient’s diary (prepared by researchers and returned upon completion of the intervention) will be conducted to assess compliance with the study. Based on previously published trials [11, 13, 14], it seems appropriate to consider those participants receiving < 75% of the recommended doses as being non-compliant.

### Concomitant medications

The physician may consider discontinuation or modification of the UTI prophylaxis if needed.

### Outcome measures

The primary outcome measures will be the frequency of UTIs during the intervention and during the 9 months period after the intervention. The secondary outcome measures are as follows: the frequency of hospitalization due to UTI, the number of days of antibiotic therapy due to UTI.

### Participant timeline

The plan for the recruitment, intervention, evaluation and visits of participants is presented in Table 1.

**Table 1:** Timetable of activities planned during the study

		Study period				
	Enrolment and allocation	Post allocation				Close-out (after the end of follow-up period)
Time point	Day 1	Month 3	Month 6	Month 9	Month 12	
Enrolment						
Eligibility screen	X					
Informed consent	X					
Randomisation of the participant	X					
Study product distribution	X					
Interventions						
Probiotic						
Placebo						
Assessments						
Recurrence of UTI		X	X	X	X	X
Frequencies of hospitalization due to UTI		X	X	X	X	X
No of days of antibiotic therapy due to UTI		X	X	X	X	X
Return of patient’s diary		X				
Return of non-used study products		X				
Telephone contact*			X	x	X	X

*UTI* Urinary tract infection

### Sample size

Based on data from previous studies [1–3], we assumed that the frequency of reccurences of UTIs will be 30% in the group of patients at similar age in one year. 88 patients are required to have a 90% chance of detection, as significant at the 5% level, a decrease in the primary outcome measure from 30% in the control group to 5% in the experimental group. Taking into account that 20% of the patients will be lost for follow-up, we have calculated that a total of 106 children will be needed.

A power and sample size calculator for the binary outcome superiority trial was used to estimate the study and control group.

### Recruitment

The recruitment rates will be followed up monthly. Patient, recruiting clinician, the centre and the trial design will be evaluated in the event of slow and poor recruitment due to reasons at various levels.

### Sequence generation

Randomisation list will be generated by the independent reasercher from Medical University of Warsaw. Block randomisation will be used, with a block size of 6. Randomisation codes will be revealed when all data will be collecting and final analysis will performed. Reaserchers and participants will not know the assignment to the group of patients during the study.

### Allocation concealment

Allocation concealment will be processed with use of opaque, sealed, numbered envelopes. It will be implemented after getting informed consent and after entering essential, demografic information to the case report form (CRF). An independent person will assign the numbered study products in accordance with randomisation list generated by a computer.

### Blinding

The probiotic and placebo will be packaged in identical sachets. Powder will look and taste similar. The sachets will be delivered by Miralex in sealed and sequentially numbered opaque envelopes. The intervention will be blinded for all participants and investigators by the end of the study.

### Data collection and management

All participants will be ensured about data confidentiality during workshop process. All study participants will be allocated to a study identification number. Data will be collected and stored in the electronic database protected by password. Only involved researchers will have access to all participants records, CFRs, all documents, laboratory data, etc.

### Statistical analysis

Intension-to treat (ITT) analysis will be performed, including all randomly assigned participants whom outcomes will be approachable (including dropouts and withdrawals). A per protocol analysis on the primary and secondary outcomes will be processed. This analysis will include children who have completed the entire treatment protocol as originally planned, with availability 9 months after intervention. X^2^ tests (Pearson's or Fisher's test) will be performed for binary outcome measures.

### Harms

Although the occurrence of adverse events as a result of participation in the current trial is not expected, data on adverse events data will be collected. All serious adverse events will be immediately reported to the project leader who will be responsible for notifying the Ethics Committee, all participating investigators and the manufacturer of the study products.

### Auditing

Auditing for this study was not required by the Bioethics Committee.

### Ethics and dissemination

The Bioethics Committee of the Medical University of Warsaw reviewed and accepted the study protocol and template consent. If any modifications to the protocol have influence on the conducting of the study, they will be presented to the Committee. Verbal and written information about informal consent will be revealed to the caregivers. The informed consent forms will have to be singed by a parent or legal gardian prior to the randomisation. Patients may abandon from the study at any time without warring, as is documented and explained at the time of providing consent. The full protocol will be available freely due to open access publication. Abstracts will be submitted to relevant national and international conferences.

## Discussion

A precise clinical question has been posed to fill a gap in knowledge as to whether administration of *L. rhamnosus PL1* and *L. plantarum PM1* are effective in the prevention of UTIs in children. The findings of this RCT, whether positive or negative, will contribute to the formulation of further recommendations on prevention of UTIs.

## Data Availability

The datasets use and analysed during the current study will be available from the corresponding author on reasonable request. All data generated and analysed during this study will be published in the article after the completed study.

## Abbreviations

UTI: Urinary tract infection
RCT: Randomised controlled trial
CAKUT: Congenital anomalies of kidney and urinary tract
VUR: Vescioureteral reflux
TMP/SMX: Trimethoprim and sulfamethoxazole
*L. rhamnosus PL1*: *Lactobacillus rhamnosus PL1*
*L. plantarum PM1*: *Lactobacillus plantarum PM1*
CRF: Case report form
ITT: Intention-to-treat

## References

[1] Simões Silva AC, Oliviera EA (2015). Update on the approach of urinary tract infection in childhood. J Pediatr (Rio J).

[2] Hellström A, Hanson E, Hansson S, Hjälmås K, Jodal U (1991). Association between urinary symptoms at 7 years old and previous urinary tract infection. Arch Dis Child.

[3] Conway PH, Cnaan A, Zaoutis T, Brandon VH, Grundmeier RW, Keren R (2007). Recurrent urinary tract infections in children risk factors and association with prophylactic antimicrobials. JAMA.

[4] National Institute for Health and Care Excellence. Urinary tract infections in children and young people. 2013. www.nice.org.uk/guidance/qs36.

[5] Stein R, Dogan HS, Hoebeke P, Kočvara R, Nijman RJ, Radmayr C, Tekgül S, European Association of Urology, European Society for Pediatric Urology (2015). Urinary tract infections in children: EAU/ESPU guidelines. Eur Urol.

[6] Żurowska A, Wasilewska A, Jung A, Kiliś-Pstrusińska K, Pańczyk-Tomaszewska M, Sikora P, Tkaczyk M, Zagożdżon I (2016). Zalecenia Polskiego Towarzystwa Nefrologii Dziecięcej dotyczące postępowania z dzieckiem z zakażeniem układu moczowego. Forum Medycyny Rodzinnej.

[7] Subcommittee on Urinary Tract Infection (2016). Reaffirmation of AAP clinical practice guideline: the diagnosis and management of the initial urinary tract infection in febrile infants and young children 2–24 months of age. Pediatrics.

[8] Garin EH, Olavarria F, Garcia Nieto V, Valenciano B, Campos A, Young L (2006). Clinical significance of primary vesicoureteral reflux and urinary antibiotic prophylaxis after acute pyelonephritis: a multicenter, randomized, controlled study. Pediatrics.

[9] Amdekar S, Singh V, Singh DD (2011). Probiotic therapy: immunomodulating approach toward urinary tract infection. Curr Microbiol.

[10] Lee SJ, Cha J, Lee JW (2016). Probiotics prophylaxis in pyelonephritis infants with normal urinary tracts. World J Pediatr.

[11] Mohseni MJ, Aryan Z, Emamzadeh-Fard S, Paydary K, Mofid V, Joudaki H, Kajbafzadeh AM (2013). Combination of probiotics and antibiotics in the prevention of recurrent urinary tract infection in children. Iran J Pediatr.

[12] Tewary K, Narchi H (2015). Recurrent urinary tract infections in children: preventive interventions other than prophylactic antibiotics. World J Methodol.

[13] Lee SJ, Shim YH, Cho SJ, Lee JW (2007). Probiotics prophylaxis in children with persistent primary vesicoureteral reflux. Pediatr Nephrol.

[14] Lee SJ, Lee JW (2015). Probiotics prophylaxis in infants with primary vesicoureteral reflux. Pediatr Nephrol.

[15] Reid G, Bruce AW (2006). Probiotics to prevent urinary tract infections: the rationale and evidence. World J Urol.

[16] Grin PM, Kowalewska PM, Alhazzan W, Fox-Robichaud AE (2013). Lactobacillus for preventing recurrent urinary tract infections in women: meta-analysis. Can J Urol.

[17] Ng QX, Peters C, Venkatanarayanan N, Goh YY, Ho CYX, Yeo WS (2018). Use of *Lactobacillus* spp. to prevent recurrent urinary tract infections in females. Med Hypotheses.

[18] Schwenger EM, Tejani AM, Loewen PS (2015). Probiotics for preventing urinary tract infections in adults and children. Cochrane Database Syst Rev.

[19] Hosseini M, Yousefifard M, Ataei N, Oraii A, Mirzay Razaz J, Izadi A (2017). The efficacy of probiotics in prevention of urinary tract infection in children: a systematic review and meta-analysis. J Pediatr Urol.

[20] Gupta V, Nag D, Garg P (2017). Recurrent urinary tract infections in women: How promising is the use of probiotics?. Indian J Med Microbiol.

[21] Rodríguez JM (2014). The origin of human milk bacteria: is there a bacterial entero-mammary pathway during late pregnancy and lactation?. Adv Nutr.

[22] Bouchard DS, Seridan B, Saraoui T, Rault L, Germon P, Gonzalez-Moreno C, Nader-Macias FME, Baud D, François P, Chuat V, Chain F, Langella P, Nicoli J, Loir YL, Even S (2015). Lactic acid bacteria isolated from bovine mammary microbiota: potential allies against bovine mastitis. PLoS ONE.

[23] Cerbo A, Palmieri B, Aponte M, Morales-Medina JC, Iannitti T (2016). Mechanisms and therapeutic effectiveness of lactobacilli. J Clin Pathol.

[24] Manzoor A, Ul-Haq I, Baig S, Qazi JI, Seratlic S (2016). Efficacy of locally isolated lactic acid bacteria against antibiotic-resistant uropathogens. Jundishapur J Microbiol.

[25] Salvetti E, O'Toole PW (2017). The genomic basis of lactobacilli as health-promoting organisms. Microbiol Spectr.

[26] Salah RB, Trabelsi I, Hamden K, Chouayekh H, Bejar S (2013). *Lactobacillus plantarum* TN8 exhibits protective effects on lipid, hepatic and renal profiles in obese rat. Anaerobe.

[27] Sadeghi-bojd S, Naghshizadian R, Mazaheri M, Ghane Sharbaf F, Assadi F (2019). Efficacy of probiotic prophylaxis after the first febrile urinary tract infection in children with normal urinary tracts. J Pediatric Infect Dis Soc.

[28] Beerepoot MA, ter Riet G, Nys S, van der Wal WM, de Borgie CA, de Reijke TM (2012). Lactobacilli versus antibiotics to prevent urinary tract infections: a randomized, double-blind, noninferiority trial in postmenopausal women. Arch Intern Med.

[29] Urinary tract infection (recurrent): antimicrobial prescribing NICE guideline. 2018. www.nice.org.uk/guidance/ng112. 10.1136/archdischild-2025-328991 41033747

